# Case of gallstone ileus and dislodged stent post endoscopic gallbladder drainage

**DOI:** 10.1055/a-2820-3915

**Published:** 2026-03-16

**Authors:** Anson Huen Yan Chan, Jacquelyn Chi Ying Fok, Stephen Ka Kei Ng, Shannon Melissa Chan

**Affiliations:** 1 13621 Division of Upper Gastrointestinal and Metabolic Surgery, Department of Surgery, Prince of Wales Hospital, Hong Kong, China; 2 26451 Faculty of Medicine, The Chinese University of Hong Kong, Hong Kong, China

Endoscopic gallbladder drainage (EGBD) is a novel technique that allows biliary drainage by deploying a stent from the gallbladder to the gastrointestinal tract under endoscopic ultrasound guidance. It has become widely accepted for biliary drainage in non-surgical candidates with acute cholecystitis 1 . While this technique is reported to have a high success rate and low adverse events profile 2 , it is not without its risk. This is the first report of a case of gallstone ileus and dislodged stent in a patient post-EGBD necessitating major laparotomy.

A 78-year-old man was first presented to us for acute calculous cholecystitis. He had past medical history of diabetes mellitus, hyperlipidaemia and ST-elevation myocardial infarction and was wheelchair-bounded. EGBD ( Video 1 ) was performed, and the patient was subsequently discharged 6 days later. He refused interval cholecystectomy due to his premorbid status.

This video illustrates a case of endoscopic gallbladder drainage in a patient with cholecystitis and the rare complication of gallstone ileus and dislodged metallic stent necessitating laparotomy.Video 1

He was admitted to us again later for intestinal obstruction. Computed tomography (CT) of the abdomen and pelvis showed dilated small bowels with an impacted 4 cm gallstone at mid jejunum causing abrupt transition. The lumen apposing metallic stent and double pigtail were also dislodged to the hepatic flexure of the colon.

**Fig. 1:**
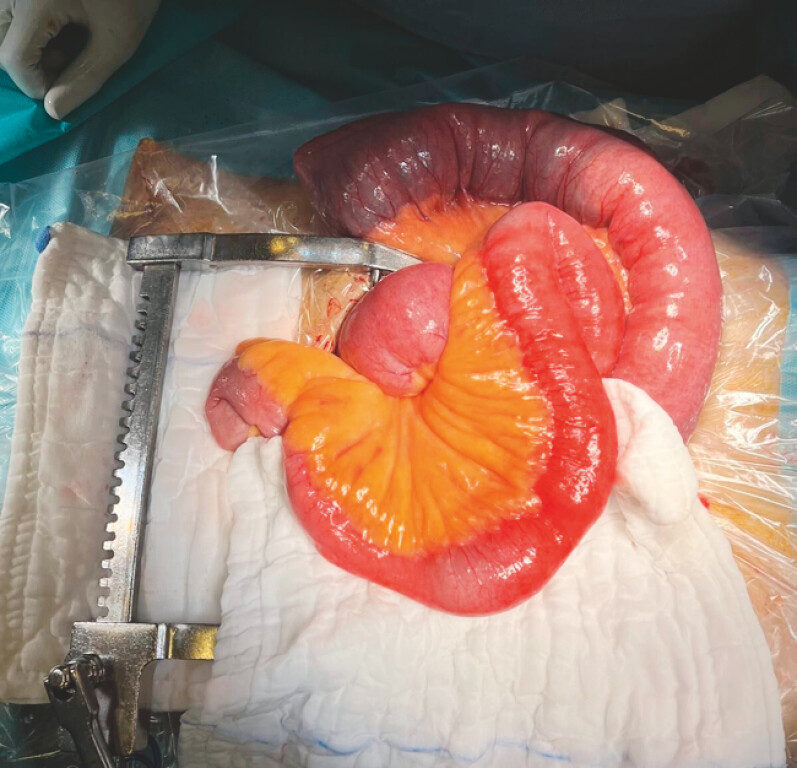
Gallstone ileus in patient post-endoscopic gallbladder drainage requiring emergency laparotomy.

**Fig. 2:**
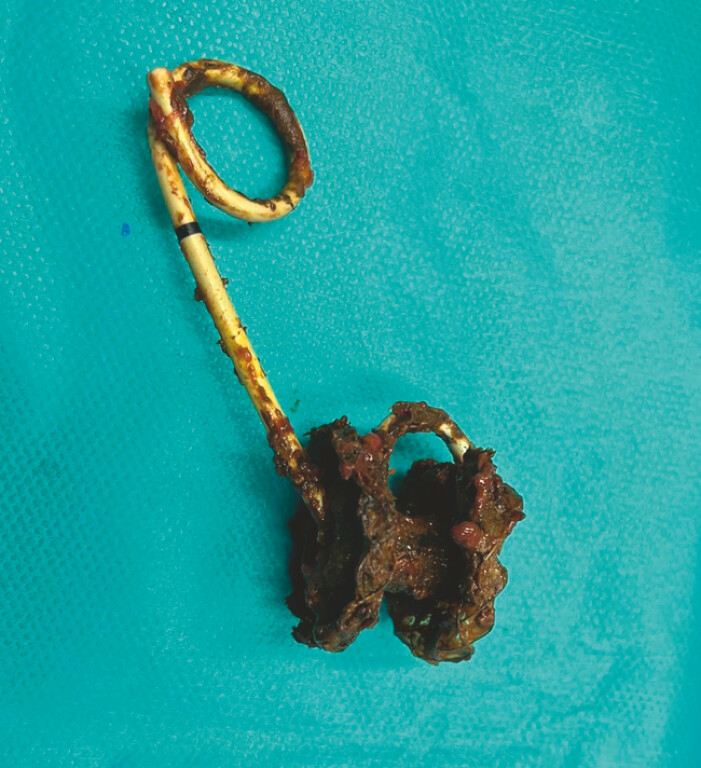
Dislodged lumen apposing metallic stent and double pigtail set retrieved via colotomy at the hepatic flexure.

Emergency laparotomy was performed with the gallstone removed through enterotomy at jejunum ( Fig. 1 ). The lumen apposing metallic stent and double pigtail were also removed through colotomy at the hepatic flexure ( Fig. 2 ). Post-operatively, the patient recovered from prolonged ileus and hospital acquired pneumonia and was discharged on post-operative day 34.

This is the first report of gallstone ileus and dislodged stent in a patient post-EGBD necessitating major laparotomy. Although rare, it remains a potential complication of EGBD with the iatrogenic cholecystoduodenal fistula. This case highlights the importance of early recognition of such a potential complication for timely surgical intervention.

Endoscopy_UCTN_Code_CPL_1AK_2AD
